# Alterations in the structural characteristics of rectus abdominis muscles caused by diabetes and pregnancy: A comparative study of the rat model and women

**DOI:** 10.1371/journal.pone.0231096

**Published:** 2020-04-03

**Authors:** Giovana Vesentini, Angélica M. P. Barbosa, Débora C. Damasceno, Gabriela Marini, Fernanda Piculo, Selma M. M. Matheus, Raghavendra L. S. Hallur, Sthefanie K. Nunes, Bruna B. Catinelli, Claudia G. Magalhães, Roberto Costa, Joelcio F. Abbade, José E. Corrente, Iracema M. P. Calderon, Marilza V. C. Rudge, The DIAMATER Study Group

**Affiliations:** 1 Perinatal Diabetes Research Center, University Hospital, Botucatu Medical School, Univ Estadual Paulista_UNESP, Botucatu, São Paulo, Brazil; 2 Department of Gynecology and Obstetrics, São Paulo State University (UNESP), Botucatu Medical School, Botucatu, São Paulo, Brazil; 3 Department of Physiotherapy and Occupational Therapy, São Paulo State University (UNESP), School of Philosophy and Sciences, Marilia, São Paulo, Brazil; 4 Department of Health Sciences, Universidade Sagrado Coração, Bauru, São Paulo, Brazil; 5 Department of Anatomy, São Paulo State University (UNESP), Institute of Biosciences, Botucatu, São Paulo, Brazil; 6 Department of Biostatistics, São Paulo State University (UNESP), Bioscience Institute, Botucatu, São Paulo, Brazil; National University Singapore Yong Loo Lin School of Medicine, SINGAPORE

## Abstract

**Background and objective:**

In the present study, we compared the effect of diabetic pregnancy on the rectus abdominis muscle (RAM) in humans and rats. We hypothesized that our animal model could provide valuable information about alterations in the RAM of women with Gestational Diabetes (GDM).

**Method:**

Newborns female rats (n = 10/group) were administered streptozotocin (100 mg/kg body weight) subcutaneously and were mated on reaching adulthood, to develop the mild hyperglycemic pregnant (MHP) rat model. At the end of pregnancy, the mothers were sacrificed, and the RAM tissue was collected. Pregnant women without GDM (non-GDM group; n = 10) and those diagnosed with GDM (GDM group; n = 8) and undergoing treatment were recruited, and RAM samples were obtained at C-section. The RAM architecture and the distribution of the fast and slow fibers and collagen were studied by immunohistochemistry.

**Results:**

No statistically significant differences in the maternal and fetal characters were observed between the groups in both rats and women. However, significant changes in RAM architecture were observed. Diabetes in pregnancy increased the abundance of slow fibers and decreased fast fiber number and area in both rats and women. A decrease in collagen distribution was observed in GDM women; however, a similar change was not observed in the MHP rats.

**Conclusion:**

Our results indicated that pregnancy- associated diabetes- induced similar structural adaptations in the RAM of women and rats with slight alterations in fiber type number and area. These findings suggest that the MHP rat model can be used for studying the effects of pregnancy-associated diabetes on the fiber structure of RAM.

## Introduction

Diabetes mellitus (DM) is a global health concern. Women who develop gestational diabetes (GDM) are more susceptible to develop type 2 diabetes later in life [1]. Pregnancy causes an insulin resistance state. When an increase in insulin secretion cannot meet the needs of the pregnancy-induced insulin resistance status, it results in the development of GDM [2]. DM is defined as a group of metabolic diseases associated with a hyperglycemic state due to metabolic or genetic malfunction in insulin release [3]. DM has also been associated with diabetic myopathy, a deficiency of healthy muscle maintenance [4]. Diabetic myopathy is a universal complication of diabetes and is related to the loss of muscle mass and strength (i.e., sarcopenia and dynapenia) [4–6].

The skeletal muscle is a heterogeneous tissue composed of different fiber types, all of which are characterized by myosin heavy chain isoforms [7]. The mammalian skeletal muscle is composed of two major fiber types—slow and fast- which differ in their size, metabolism, and contractile properties [8]. Another component of skeletal muscle is the extracellular matrix (ECM), which plays major roles in muscle fiber force transmission, maintenance, and repair [9]. The skeletal muscle is known to play a critical role in locomotion and glucose homeostasis [10].

Although significant improvements have been made over the past decade in the care and management of GDM with respect to adverse pregnancy outcomes [11, 12], there are only a few studies on the impact of GDM on urinary disorders such as urinary incontinence (UI). Data from a previous study suggested that up to 49% of women with GDM have a substantial risk of developing UI [13]. The consequences of UI persist not only during pregnancy but up to 2 years post-partum and have a negative impact on the quality of life [14, 15]. Previously, our research group conducted studies on the urethral tissue obtained from a pregnant Streptozotocin (STZ) rat model [16–21]. The hyperglycemia status in these rats is manifested by STZ-induced necrosis of the pancreatic β-cells [22]. However, the individual animals, dose, route of administration, and life period of induction are key factors contribuiting to the intensity of the induced hyperglycemia [16]. A higher or lower hyperglycemic level caused an impairment of the urethral tissue [17, 18, 20]. We also investigated the changes in rectus abdominis muscle (RAM) in the same animal model and discovered that both the RAM and urethral muscles are subjected to similar morphological changes during diabetic myopathy [21].

The RAM is a typical glycolytic muscle with a predominance of fast fibers [21]. The increase in its abdominal content during pregnancy represents a chronic physiological stimulus in the muscle fibers of the abdominal wall [23]. During pregnancy, there is an increase in the abundance of slow fibers in the RAM, enabling it to be stretched [21, 23]. This stretching is hypothesized to be a result of an overload of hypertrophy, contributing to muscle tone and endurance [23]. There is growing scientific and clinical attention on the role of the abdominal muscles in the normal functioning of the pelvic floor muscle in women [24]. Changes in the structure of these muscles may jeopardize their support and continence [25].

Although previous studies have demonstrated the relationship between UI and GDM [13–15], the pathophysiology of GDM leading to the development of UI is poorly understood. Investigation of the human urethra or pelvic floor muscles have many ethical constraints, and the studies on human UI predominantly rely on indirect assessments via clinical examination or imaging techniques [26–28]. However, there are several unanswered questions regarding the pathogenesis of urinary disorders. Rodent models can be used as representative animal models to determine the possible events leading to the high prevalence of UI in women with GDM. The structure and histology of the abdominal wall muscles of rats are well characterized and are similar to those in humans, making them appropriate tissue models for studying the physiological changes in the muscle [29].

The purpose of this study is to compare the effects of diabetes and pregnancy in human and rodent RAM using histological and immunohistochemical techniques to elucidate the suitability of the rat model for studying the pathophysiology of human GDM-induced UI. This study provides a foundation for the use of the rat model for studying diabetic myopathy in humans as a reliable tool for future studies on GDM and the development of new therapeutic approaches.

## Materials and methods

### Ethics statement

All animal experiments were approved by the Institutional Animal Care and Use Committee, Faculdade de Medicina de Botucatu, São Paulo State University (UNESP), and complied with the applicable regulations and recommendations of the Brazilian authorities (protocol 1003–2013).

For human study, signed informed consent was obtained from all study participants before the start of the study. Participants were recruited at the University Hospital (Perinatal Diabetes Research Center), UNESP, Brazil, between March 2015 and December 2018. The study was registered in the Brazilian National Research Registry platform (Plataforma Brasil) and approved by the National Committee for Ethics in Research (CONEP) (CAAE: 26142614.0.0000.5411 and CAAE: 82225617.0.0000.5411) and adhered to the guidelines of the Declaration of Helsinki on Human Experimentation.

### Animal model

Female and male Wistar (12–13 weeks-old and 250–300 g) rats were obtained from the Multidisciplinary Center for Biological Investigation (Campinas, SP, Brazil). Animals were housed in a facility with constant temperature (22±2°C) and humidity (55±5%) on a controlled 12 h light–12 h dark cycle with food and water *ad libitum*. After one week of acclimatization, the dams were mated. The female offspring, on the first day of life, were randomly assigned to two groups (n = 10/group) the mild hyperglycemic pregnant (MHP) group, which received STZ (SIGMA Chemical Company, St. Louis, MO, USA), diluted in 0.1 M citrate buffer (pH 4.5) at a dose of 100 mg/kg by subcutaneous injection [30], or the non-mild hyperglycemic pregnant (non-MHP) group, which received the same dose of citrate buffer. When these rats reached adulthood (around 12–13 weeks- old), they were housed with adult male rats overnight. The first day of gestation (GD0) was determined by examining the vaginal smear, and the rats were housed in individual cages after that. An oral glucose tolerance test (OGTT) was performed on the 17^th^ day of pregnancy to assess the development of altered glucose metabolism [31]. Blood glucose concentrations were measured using a One-Touch Ultra glucometer (LifeScan, Johnson and Johnson®, Milpitas, CA, USA), and the values were expressed as mg/dL. At the end of pregnancy (GD21), the dams were euthanized by sodium thiopental injection (Thiopentax®, Brazil 80 mg/kg dose). The lower third of RAM was exposed, dissected, and removed. The edges were reduced, and the sample was wrapped in talc, frozen in liquid nitrogen, and kept at -80°C. About 500 mg non-random samples of RAM were obtained from a total of 10 rats in each group. The morphometric and immunohistochemical data from the maternal and fetal samples were published previously by Vesentini et al. [21].

### Participant selection

**Fig 1:**
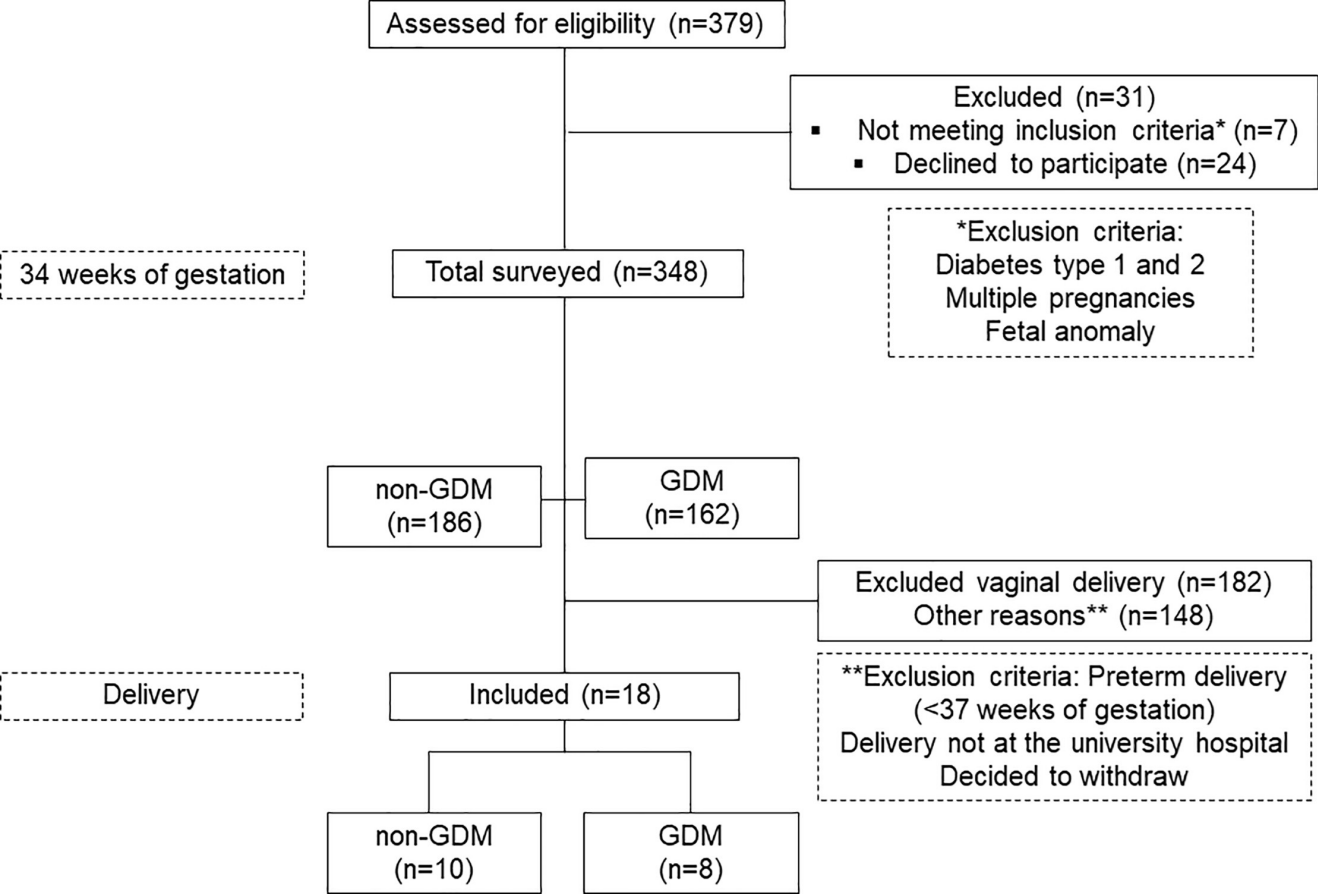
Flowchart of participant recruitment strategy.

Pregnant women were screened for GDM between 24–28 weeks of gestation and were diagnosed according to the ADA criteria using a 75 g-OGTT test [32, 33]. Women with known type 1 or type 2 DM, preterm delivery (<37 weeks of gestation), multiple pregnancies, or known fetal anomaly were excluded. All women with GDM underwent the same treatment in the Perinatal Diabetes Research Center (PDRC). This treatment protocol included adequate nutrition based on recommendations from a nutritionist, motivation to exercise regularly, and insulin administration. Participants with singleton pregnancies who were screened for GDM and met the inclusion criteria were invited at 34 weeks of pregnancy. Around 500 mg of non-random sampling of RAM was obtained from a total of 18 pregnant women who underwent C-section and were categorized into the non-GDM group (n = 10) or GDM group (n = 8) (Fig 1). RAM biopsies were obtained at the time of C-section within 10 min of delivery. The sample was stripped off from visible adipose and connective tissues, wrapped in talc, snap-frozen in liquid nitrogen, and stored below -80°C.

### Histological examination, immunohistochemical staining, and morphometric analysis

Both the rat and human muscle samples were processed similarly. Muscle samples were cut into 10-μm-thick cross-sections using a cryostat (Leica CM 1800). The cross-sections were fixed on a microscope glass slides in cold acetone for 10 minutes and were stained using hematoxylin and eosin (H&E) and picrosirius red, or were processed for immunohistochemical analyses. The slides were examined by light microscopy and photographed (DMR, Leica® coupled with a CCD-IRIS/RGB digital camera, Sony®). The micrographs published by Vesentini et al. [21] previously were re-analyzed, and the morphometric area of the fiber types and collagen and fiber numbers were determined.

Picrosirius red staining was performed to determine the tissue area of collagen (red-stained). For quantitative morphometric analysis, ten sections were stained forcollagen area and imaged under 20× magnification. The images were analyzed using ImageJ (National Institutes of Health, USA).

For immunohistochemistry of fast and slow-type skeletal muscle fibers, the sections were incubated with antibodies directed against WB-myosin heavy chain, fast (WB-MHCf) Novocastra (rats, 1:120; human, 1:160) and WB-MHC slow (WB-MHCs) Novocastra (rats, 1:180; human, 1:120). The fiber type area and number were quantitatively determined, as described previously [21].

### Statistical analysis

Categorical data were described by percentages and assessed by chi-square tests. Continuous data were described by their means ± standard deviations (SD) and compared by *t*-tests (clinical characteristics of participants, rats fetal weight, fiber area), ANOVA (rat maternal weight at day 0 and 21), or Poisson test (fiber type number). The OGTT results were calculated using the total area under the curve (AUC) [34] and compared by *t*-tests. Statistical significance was set as a *p*-value < 0.05. All analyses were performed using SAS for Windows, v.9.3 (Statistical Analysis System Institute Inc., USA).

## Results

**Fig 2:**
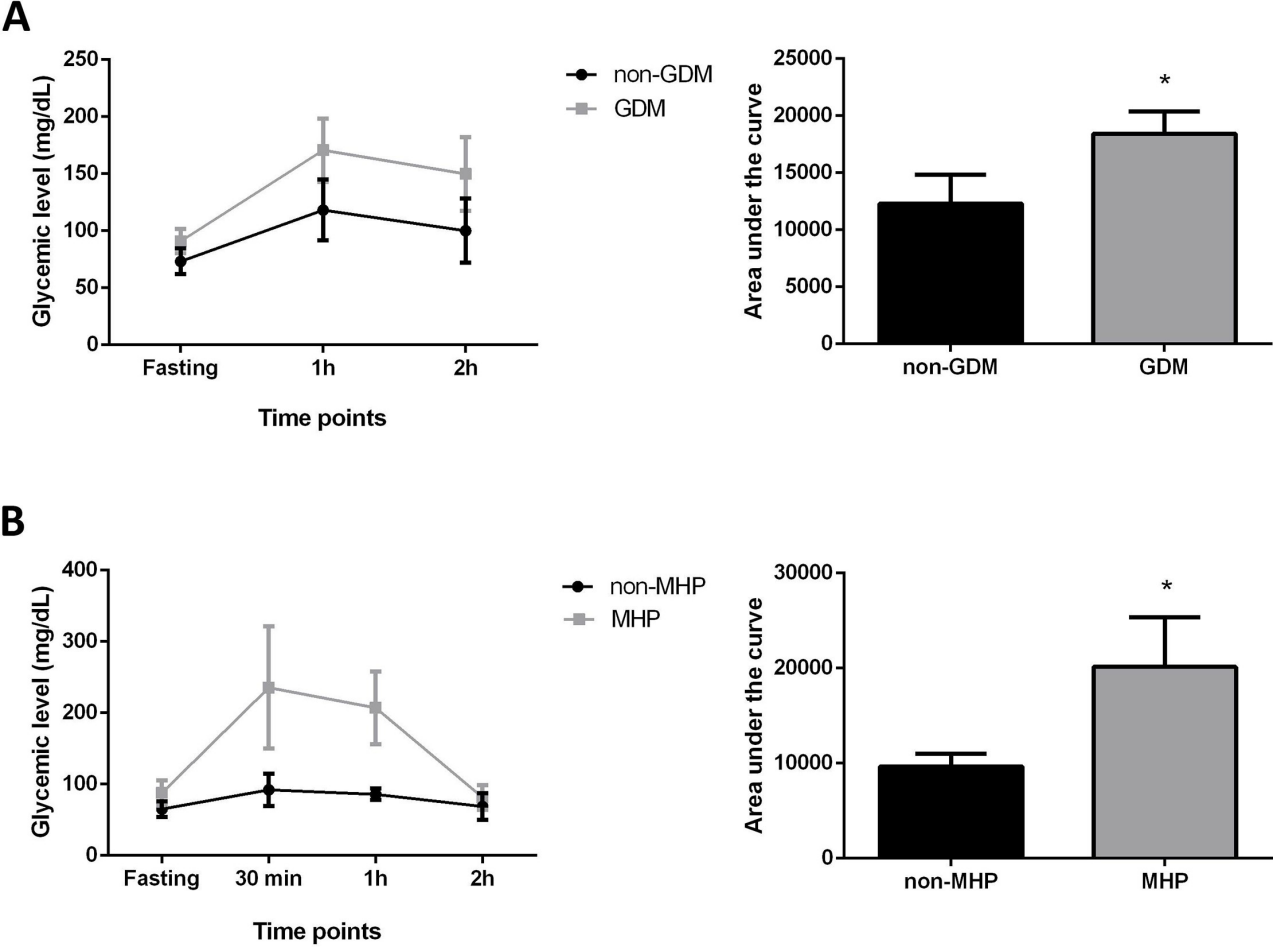
A: Oral Glucose Tolerance Test (OGTT) performed at 24–28 weeks for pregnant women and on the 17^th^ day of pregnancy for rats. B: The area under the curve of each group is expressed as the mean ± standard deviation. *p<0.05 shows a significant difference compared to the control group (*t*-test).

Table 1 displays the socio-demographic and clinical characteristics of the study participants. No statistically significant differences were observed between the groups regarding any of the variables. Table 2 shows the maternal and fetal weight of the rats in the two groups. The absence of any significant differences highlights the homogeneity of the samples in both the pregnant women and rats. The GDM and MHP groups, presented higher AUC with elevated glucose levels compared to control groups (non-GDM = 12303.3 ± 2547.6 mg/dL X minutes; GDM = 18428.6 ± 1963.2 mg/dL X minutes, *p*<0.0001; non-MHP = 9662.5 ± 1339.2 mg/dL X minutes; MHP = 20142 ± 5194.6 mg/dL X minutes, *p* = 0.001) (Fig 2).

**Table 1:** Socio-demographic and clinical characteristics of the study participants.

	non-GDM (n = 10) Mean (SD)	GDM (n = 8) Mean (SD)	*p*-value
Age (years)	29.80 (5.03)	34.50 (6.07)	0.10
HbA1c	5.31 (0.66)	5.36 (0.34)	0.08
**Parity (%)**			
**Nulliparous**	20%	50%	0.09
**Multiparous**	80%	50%	
Prepregnancy BMI (kg/m^2^)	33.63 (7.55)	29.91 (5.25)	0.30
BMI at the end of gestation (kg/m^2^)	38.22 (6.26)	34.08 (4.30)	0.14
Weight gain during pregnancy (kg)	12.11 (7.96)	10.46 (6.01)	0.52
**Ethnicity**			
White (%)	6 (60%)	5 (62.5%)	0.40
**Educational level**			
Primary	4 (40%)	3 (37.5%)	0.32
High school	4 (40%)	4 (50%)	
University degree	2 (20%)	1 (12.5%)	
**Hypertension**			
Yes	5 (50%)	1 (12.5%)	0.22
Newborn weight (g)	3408 (269.92)	3555 (335.4)	0.45

Data presented as number (%) or mean ± standard deviation. Abbreviations: SD, standard deviation; BMI, body mass index. *p<0.05 shows a significant difference compared to the control group.

**Table 2:** Maternal and fetal weights in the animal study groups.

	non-MHP (n = 10) Mean (SD)	MHP (n = 10) Mean (SD)	*p*-value
Maternal weight on day 0 (g)	257.33 (18.49)	254.53 (22.57)	0.99
Maternal weight on day 21 (g)	374.61 (30.58)	349.39 (38.03)	0.05
Fetal weight (g)	5.46 (0.58)	5.48 (0.61)	0.77
HbA1c	3.3 (0.82)	3.74 (1.92)	0.65

Data presented as mean ± standard deviation. Abbreviations: SD, standard deviation. *p<0.05 shows a significant difference compared to the control group.

**Fig 3:**
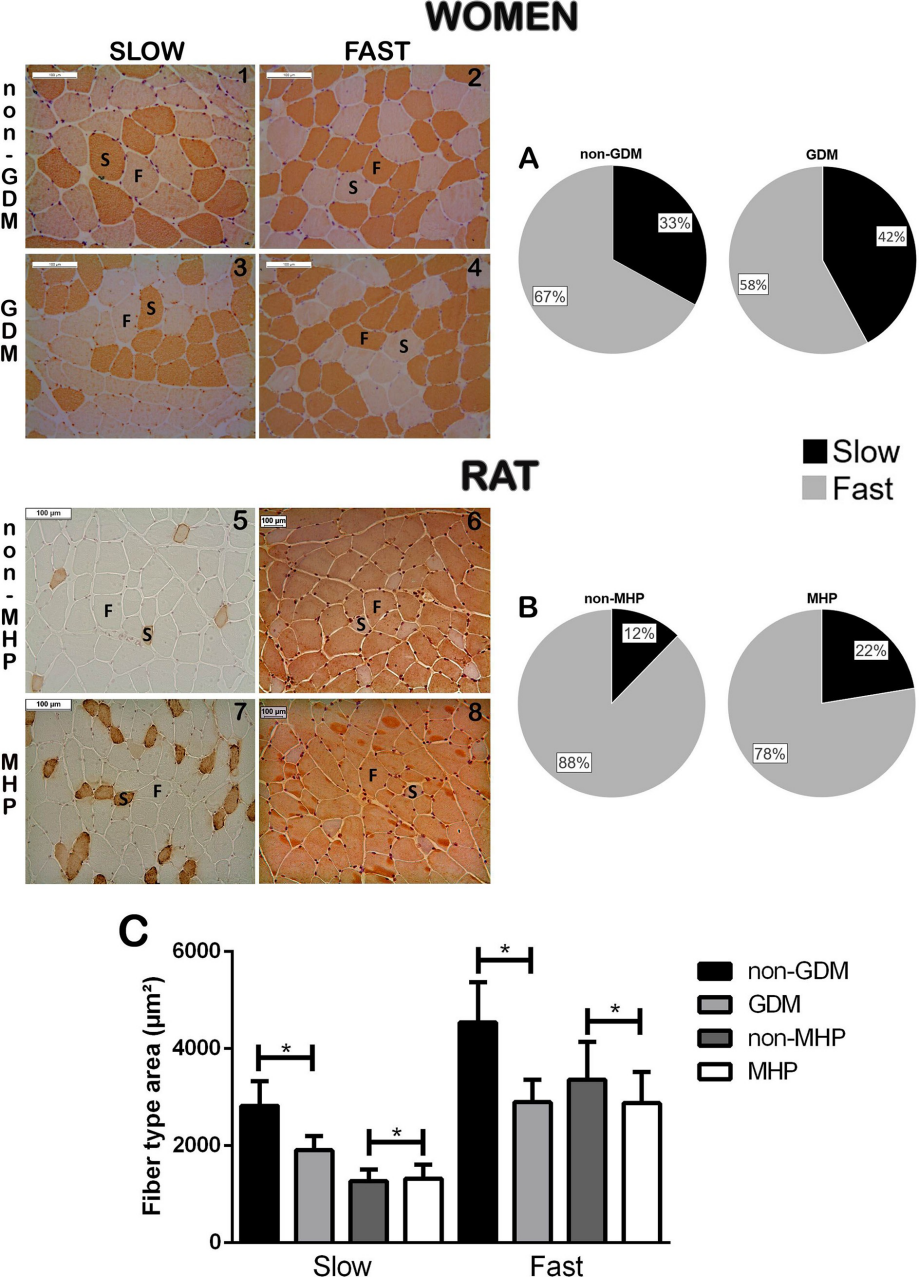
Micrographs showing slow and fast fibers in a transverse RAM section. Non-GDM (slow 1, fast 2), GDM (slow 3, fast 4), non-MHP (slow 5, fast 6), and MHP (slow 7, fast 8). (A) The abundance of each fiber type is expressed as percentages, and (B) the area of each fiber type is expressed as mean ± SD. Differences in the abundance of each fiber type between the groups were determined using Poisson distribution. Differences in the fiber area between the groups were determined using the Student’s *t*-test. *p<0.05 shows a significant difference compared to the control group. Abbreviations: GDM, Gestational Diabetes, MHP, mild hyperglycemic pregnant.

Immunohistochemical analysis of the markers of fast and slow type skeletal muscle fibers during pregnancy in the non-GDM and non-MHP groups showed an increased abundance of fast fibers in the RAM (Fig 3). Despite the higher number of fast fibers (non-GDM = 66.98 ± 10.75%; GDM = 57.93 ± 8.22, p = 0.0012; non-MHP = 87.71 ± 6.24%; MHP = 77.58 ± 6.63, p<0.0001), a significant increase in the number of slow fibers (non-GDM = 33.02 ± 15.32%; GDM = 42.07 ± 9.65, p<0.0001; non-MHP = 12.29 ± 16.69%; MHP = 77.58 ± 6.63, p<0.0001) was observed both in the GDM and MHP groups compared to that in the respective controls. The GDM and MHP groups showed a decrease in the area of fast fibers (non-GDM = 4544.82 ± 825.54 μm^2^; GDM = 2895.8 ± 459.2 μm^2^, *p*<0.0001; non-MHP = 3363.29 ± 773.51 μm^2,^ MHP = 2878.35 ± 640.3 μm^2^, *p*<0.0001). The distribution of the slow fiber area presented different patterns in the two groups. While in the GDM group (human) there was a decrease in the slow fiber area (non-GDM = 2820.89 ± 509.23; μm^2^; GDM = 1908.3 ± 294.3 <m^2^, *p*<0.0001), an increase (non-MHP = 1273.63 ± 233.9 μm^2^; MHP = 1324.85 ± 286.46 μm^2^, *p* = 0.0178) was observed in the MHP (rat) group.

**Fig 4:**
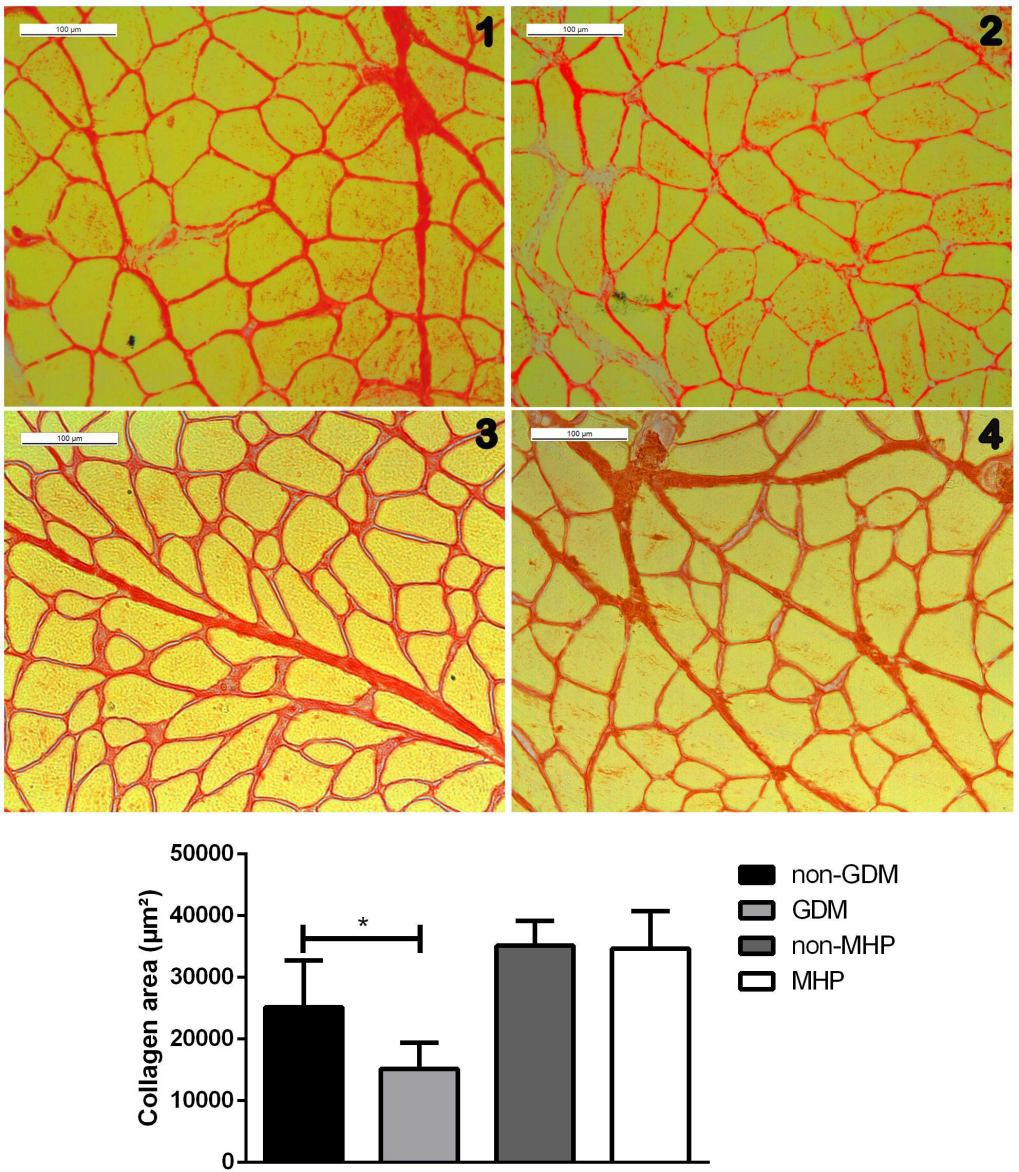
**Transverse RAM sections stained with picrosirius red showing striated muscle (yellow) and collagen (red). (1) Non-GDM, (2) GDM, (3) non-MHP, and (4) MHP.** Differences in the collagen area between the groups were evaluated using the Student’s *t*-test. *p<0.05 shows a significant difference compared to the control group. Abbreviations: GDM, Gestational Diabetes, MHP, mild hyperglycemic pregnant; RAM, rectus abdominis muscle.

The collagen area in the GDM group was significantly reduced compared to that in the non-GDM group (non-GDM = 25194.2 ± 7579.1 μm^2^; GDM = 15208.3 ± 4181.2 μm^2^, p<0.0001). On the other hand, there were no differences in the collagen area between the rat groups (non-MHP = 35150.7 ± 4010.3 μm^2^; MHP = 34701.1 ± 6078.7 μm^2^, p = 0.5376) (Fig 4).Table 3 summarizes the morphological changes of the RAM in the rats and human diabetic groups.

**Table 3:** Morphological changes in the RAM of pregnant rats and women with diabetes.

	MHP (rats) (Vesentini et al., 2018)	GDM (women)
**Collagen area**	Ns	**↓**
**Fiber type area**	↓ FAST	↓ FAST
	↑ SLOW	↓ SLOW
**Fiber type number**	↓ FAST	↓ FAST
	↑ SLOW	↑ SLOW

Abbreviations: ns, not significant

## Discussion

Increased risk of adverse pregnancy outcomes such as the increased risk of developing metabolic syndrome or diabetes, traumatic delivery complications, macrosomia, stillbirths, and congenital anomalies are associated with GDM [35–37]. The consequences of GDM for maternal and neonatal studies have been recognized for a long time [38], and as a result, treatment of GDM is primarily aimed at reducing the risk of adverse perinatal outcomes [12, 39].

The association between urinary disorders and GDM is not well understood. Urinary disorders have been a neglected aspect of GDM and are not addressed in the guidelines for the care of Gestational Diabetes [11, 40, 41]. This lack of consideration might be due to the lack of robust evidence supporting the association between GDM and urinary disorders. Few studies have pointed out the influence of GDM on skeletal muscle morphology. Given the ethical constraints associated with the use of a large amount of tissue for a comprehensive analysis of RAM in women, research involving animal models is critical to our understanding of the role of GDM in the development of urinary disorders. Therefore, the present study aimed to compare histological changes caused by diabetes and pregnancy in the RAM of humans and rats. The characteristics of muscle overload in rats (i.e., weight gain and fetal weight) and women (i.e., weight gain and baby weight) did not show any statistical differences, suggesting that the changes observed are related only to the hyperglycemic status. Previous studies showed that diabetes could cause skeletal muscle fibers to become atrophic, leading to a loss of muscle mass [42, 43].

Our findings revealed that among patterns of pregnant diabetic myopathy in rats and women, diabetes during pregnancy significantly impacted the structural characteristics of the RAM tissue. Despite this, the number of dominant fast fiber number in RAM samples was similar in women and rats, regardless of diabetes and pregnancy. Our results showed that diabetes during pregnancy modify the RAM fiber type number and decrease the fast fiber area. Moreover, in rats, no change in the collagen area was observed between the MHP and non-MHP groups. Together these findings demonstrate that RAM exposed to a diabetic environment is characterized by a decrease in the number and area of the fast fiber and an increase in the number of slow fibers. Although MHP and GDM showed similar changes in the fast fiber number, fast fiber area, and slow fiber number, the collagen area in GDM showed a decrease. Taken together, our results demonstrate that RAM is vulnerable to histological architecture changes due to GDM in humans. The alterations in the muscle fiber pattern of RAM could influence its functionality both in GDM or MHP rats.

Skeletal muscle atrophy is a complex molecular process that is not entirely understood. Reduced muscle fiber number and/or size is associated with a decrease in muscle function and can be caused by age [44], disuse [45] and illness [46]. Strong evidence suggests that diabetes is associated with muscular changes such as reduced muscle strength [47], power [48], mass [49], quality [48], and endurance and fiber type switch [6, 50] termed as diabetic myopathy [6, 51]. Our findings show that in the hyperglycemic environment, skeletal muscle in both rats and women decrease the number and area of fast fibers and an increase in the number of slow fibers. Diabetes is characterized by a fast-to-slow fiber type shift with preferential atrophy of fast glycolytic muscle fibers. The reason for the increase in the number of slow fibers in diabetes is currently unknown. Studies suggest that slow fibers have a stronger influence on muscle insulin action and glucose handling capacity [52]. This might be related to a compensatory response of skeletal muscle due to hyperglycemia to regulate metabolic homeostasis. Slow fiber type has a higher turnover of protein synthesis and degradation, an oxidative profile with larger mitochondrial content, higher myoglobin, increased insulin sensitivity, and a higher GLUT4 expression compared to fast fiber [52, 53]. Similar changes are seen in cancer cachexia [54], aging-related sarcopenia [55], and Huntington's Disease [56]. The differences in the slow fiber area between the MHP (rat) and GDM (human) groups may be due to the relatively short pregnancy time and the higher number of fetuses in the rats and high weight gain in the humans.

Collagen is the major structural component of the skeletal muscle ECM [9]. ECM is highly adaptavite and, therefore, capable of remodeling in response to physiological stimuli or disease [9]. Studies have shown that during late-pregnancy in rats, there are marked alterations in ECM components in the pelvic floor muscles [57], RAM [21], and vagina [58]. These passive mechanical structures undergo significant maternal adaptations during pregnancy in preparation for parturition and birth [59]. Previous studies show that diabetes is characterized by an increase in muscle collagen [60, 61]. According to Kang [62], the inflammatory response associated with insulin resistance has extensive effects on increased collagen deposition and ECM remodeling. Although we hypothesized that pregnancy-associated with diabetes would result in fibrosis, in our results, the distribution of muscle collagen in rats and humans showed different trends—in rats, there was no change in the collagen area while in humans, a significant decrease in collagen was observed. It is not known whether the decrease in collagen observed in our studies was due to a decrease in synthesis, an increase in collagen degradation or the excessive muscle stretching caused by pregnancy. Although pregnancy and diabetes are known to cause muscle fibrosis independent of each other, we speculate that they occur together in a muscle during pregnancy causing substantial muscle strain altering the various factors associated with collagen synthesis. However, further studies are necessary to understand the effect of diabetes and pregnancy on the distribution of collagen in the muscle fibers.

The RAM of rats during pregnancy undergo adaptations that change muscle architecture to facilitate fetal delivery [23]. However, diabetes affects the abdominal muscle substantially. Previous studies found that GDM causes alterations of important mediators of insulin resistance and inflammation [63, 64] in tissues of the abdominal wall obtained during C-section. Whether these alterations persist after delivery is unknown. These relationships deserve further attention as they may represent implications on the effectiveness of interventions on treatment and prevention of GDM consequences.

An ideal animal model for GDM research has not been established yet. Our results showing differences and similarities between GDM in humans and the MHP rats suggest that the MHP rats could be used as a preliminary prototype model for future research in the field of diabetic myopathy and the development of new therapeutical approaches. Accurate histopathological diagnosis and identification of the underlying mechanisms leading to skeletal muscle changes caused by GDM would result in a better understanding of the disease and development of new personalized patient management strategies.

One limitation of this study is the use of a quadrupedal animal model that differs from humans with respect to the effect of gravity on the biomechanics of RAM and the size and number of fetuses. However, our animal model provides the opportunity to test hypotheses more rigorously in a controlled environment. Rodents are attractive animal models because it is possible to work with large numbers of rodents in a cost-effective manner. Moreover, the pregnancy period in rodents is only 21 to 23 days [65]. In addition, the morphology and architecture of the abdominal wall of rats are similar to that of humans [29]. In this study, we present an animal model that is comparable with the glycemic levels of GDM in women and may, therefore, be applicable for future research on the molecular mechanism of GDM pathogenesis and for developing novel therapeutic approaches for GDM and UI.

## Conclusion

The present study is the first to show that RAM fast fiber predominance is preserved in GDM women and MHP rats. Furthermore, our results demonstrate that RAM slow fiber and collagen are decreased in GDM. However, no changes in collagen patterns were detected in RAM samples of MHP rats. The comparison of skeletal muscle fibers between GDM women and MHP rats revealed that both underwent similar profound architectural changes, suggesting that they might have a comparable functional change in response to diabetes and pregnancy.

## Supporting information

S1 Table Mean SD values of morphological analysis in the study (Part A). (PDF)

S2 Table Mean SD values of morphological analysis in the study (Part B). (PDF)
