# Supplementary material for: Alterations in the structural characteristics of rectus abdominis muscles caused by diabetes and pregnancy: A comparative study of the rat model and women
Source: PLoS One. 2020 Apr 3;15(4):e0231096. doi: 10.1371/journal.pone.0231096 (PMC7122752; doi:10.1371/journal.pone.0231096)
Supplement: S1 Table — (PDF) [file pone.0231096.s001.pdf]

S1 Table. Mean SD values of morphological analysis in the study (Part A).

COLLAGEN	Mean	Std Dev	Std Err	Minimum	Maximum	Mean	95% CL Mean		Std Dev	95% CL Std Dev		p-value	
non-DMG	25194.2	7579.1	757.9	13481.5	41813.9	25194.2	23690.3	26698.0	7579.1	6654.5	8804.5		
DMG	15208.3	4181.2	467.5	7014.8	22988.5	15208.3	14277.8	16138.8	4181.2	3618.6	4952.5	<.0001	
non-MHP	35150.7	4010.3	401.0	25787.0	44033.0	35150.7	34355.0	35946.5	4010.3	3521.1	4658.7		
MHP	34701.1	6078.7	607.9	24522.0	45050.0	34701.1	33494.9	35907.2	6078.7	5337.2	7061.5	0.5376	
**SLOW_AREA**													t-test
non-DMG	2820.9	509.2	184.718	1088.0	4530.2	2820.9	2784.6	2857.2	509.2	484.9	536.2		
DMG	1908.3	294.3	131.764	1118.2	2911.1	1908.3	1882.5	1934.2	294.3	277.1	313.8	<.0001	
**FAST_AREA**													
non-DMG	4544.8	825.5	284.839	2703.8	6979.0	4544.8	4488.9	4600.7	825.5	787.9	867.0		
DMG	2895.8	459.2	170.294	1877.3	3957.1	2895.8	2862.4	2929.3	459.2	436.7	484.1	<.0001	
