# Supplementary material for: Alterations in the structural characteristics of rectus abdominis muscles caused by diabetes and pregnancy: A comparative study of the rat model and women
Source: PLoS One. 2020 Apr 3;15(4):e0231096. doi: 10.1371/journal.pone.0231096 (PMC7122752; doi:10.1371/journal.pone.0231096)
Supplement: S2 Table — (PDF) [file pone.0231096.s002.pdf]

S2 Table. Mean SD values of morphological analysis in the study (Part B).

SLOW_AREA	Mean	Std Dev	Minimum	Maximum	Median	p-value	
non-MHP	1273.63	233.90	730.26	2199.88	1216.28	0.0178	Gamma
MHP	1324.85	286.46	935.78	2298.12	1241.84		
**FAST_AREA**							
non-MHP	3363.29	773.51	1275.53	6725.43	3295.40	<.0001	
MHP	2878.35	640.30	1479.62	4878.71	2829.94		
**SLOW_NUMBER**	**Estimate**	**Standard Error**	**z Value**	**Mean**	**Standard Error of Mean**	**p-value**	
non-DMG	-11.080	0.04845	-22.87	0.3302	0.01600	<.0001	Poisson
DMG	-0.8659	0.03412	-25.38	0.4207	0.01435		
non-MHP	-20.964	0.05278	-39.72	0.1229	0.006487	<.0001	
MHP	-14.952	0.03904	-38.30	0.2242	0.008753		
**FAST_NUMBER**							
non-DMG	-0.4008	0.03402	-11.78	0.6698	0.02279	0.0012	
DMG	-0.5459	0.02907	-18.78	0.5793	0.01684		
non-MHP	-0.1311	0.01976	-6.64	0.8771	0.01733	<.0001	
MHP	-0.2539	0.02099	-12.09	0.7758	0.01628		
